# A SLAF-based high-density genetic map construction and genetic architecture of thermotolerant traits in maize (*Zea mays L.*)

**DOI:** 10.3389/fpls.2024.1338086

**Published:** 2024-02-07

**Authors:** Tingting Wen, Xuefei Zhang, Jiaojiao Zhu, Susu Zhang, Mohammad Saidur Rhaman, Wei Zeng

**Affiliations:** ^1^ Peking University Institute of Advanced Agricultural Sciences, Shandong Laboratory of Advanced Agriculture Sciences in Weifang, Weifang, China; ^2^ Seed Administration Station of Shandong Province, Jinan, China; ^3^ Taian Daiyue District Bureau of Agriculture and Rural Affairs, Taian, China

**Keywords:** maize, thermotolerance, flowering, genetic map, RIL population, candidate genes

## Abstract

The leaf scorching trait at flowering is a crucial thermosensitive phenotype in maize under high temperature stress (HS), yet the genetic basis of this trait remains poorly understood. In this study, we genotyped a 254 RIL-F_2:8_ population, derived from the leaf scorch-free parental inbred line Abe2 and the leaf scorching maternal inbred line B73, using the specific-locus amplified fragment sequencing (SLAF-seq) method. A total of 10,112 polymorphic SLAF markers were developed, and a high-density genetic map with a total length of 1,475.88 cM was constructed. The average sequencing depth of the parents was 55.23X, and that of the progeny was 12.53X. Then, we identified a total of 16 QTLs associated with thermotolerant traits at flowering, of which four QTLs of leaf scorching damage (LS) were distributed on chromosomes 1 (*qLS1*), 2 (*qLS2.1*, *qLS2.2*) and 3 (*qLS3*), which could explain 19.73% of phenotypic variation. Combining one *qLS1* locus with QTL-seq results led to the identification of 6 candidate genes. Expression experiments and sequence variation indicated that *Zm00001d033328*, encoding N-acetyl-gamma-glutamyl-phosphate reductase, was the most likely candidate gene controlling thermotolerant traits at flowering. In summary, the high-density genetic map and genetic basis of thermotolerant traits lay a critical foundation for mapping other complex traits and identifying the genes associated with thermotolerant traits in maize.

## Introduction

Maize (*Zea Mays* L.), recognized as one of the most crucial food crops worldwide, plays a pivotal role in ensuring global food security and fostering sustainable agricultural development for the future (Shiferaw et al., 2011). Despite its significance, the quality and yield of maize face numerous threats from abiotic stresses, particularly in the maize-growing regions within China’s North-South climate transition zone. This transitional zone exhibits significant environmental complexity, biodiversity, and climate sensitivity, often encountering a multitude of bio-adversities and abiotic stresses, notably the high temperatures experienced during the summer months (Kou et al., 2020). Elevated temperatures exceeding 35°C can lead to severe damage in maize development, including reduced pollen vigor, prolonged anthesis-silk interval (ASI), and diminished yields (Dupuis and Dumas, 1990; Fahad et al., 2017; Lizaso et al., 2018). Despite these challenges, there has been limited research on the genetic mechanisms underlying high temperature stress (HS) during the reproductive stage of maize. The leaf scorching trait represents a critical thermosensitive phenotype during the reproductive stage of maize, yet only limited research has been undertaken in this area (Frey et al., 2016). This scarcity of studies can be attributed to the tendency of researchers to avoid hot weather during the maize reproductive stage in order to safeguard corn quality and yield. Consequently, the leaf scorching phenotype is seldom observed under normal maize growth conditions (Frey et al., 2016). Therefore, there is a compelling need for a comprehensive investigation into the leaf scorching traits during high-temperature stress at the reproductive stage of maize. This experiment aims to categorize leaf scorching traits into three indicators—Leaf Scorching damage (LS), Leaf Scorching Degree (LSD), and Leaf Scorching Ratio (LSR)—in order to explore the genetic mechanisms of thermotolerance during maize flowering.

The construction of a genetic map is an indispensable tool for analyzing genetic mechanisms and facilitating molecular marker-assisted breeding. In previous years, the utilization of simple sequence repeat (SSR) markers had been prevalent in numerous crop studies for genetic map construction. However, the limited polymorphism of SSR markers in maize posed challenges in establishing a comprehensive genetic map, thereby constraining their application in fine mapping and marker-assisted selection breeding (MAS) across various crops (Wang et al., 2019). In recent times, single nucleotide polymorphism (SNP) markers have gained popularity in genetic map construction due to their extensive variability throughout the entire genome (Wang et al., 2015). A recently developed high-throughput strategy known as Specific-Locus Amplified Fragment Sequencing (SLAF-seq) has emerged as a valuable approach for large-scale SNP development and genotyping, leveraging next-generation sequencing (NGS) technology (Yang et al., 2019).

This technology has been effectively applied to the construction of genetic maps and QTL analysis in various plants, including such as cotton (*Gossypium hirsutum*) (Huang et al., 2021), *Thinopyrum ponticum* (Liu et al., 2018), bread wheat (*Triticum aestivum*) (Yang et al., 2019), soybean (*Glycine max L. Merr.*) (Han et al., 2019), pepper (*Capsicum frutescens*) (Guo et al., 2017) (Zhang et al., 2019), black gram (*Vigna mungo (L.) Hepper*) (Somta et al., 2019), flax (*Linum usitatissimum L.*) (Wu et al., 2018), wolfberry (*Lycium Linn.*) (Zhao et al., 2019), broccoli (*Brassica oleracea L. italic*) (Yu et al., 2019), watermelon (*Citrullus Lanatus L.*) (Li et al., 2018), sunflower (*Helianthus annuus L.*) (Zhou et al., 2018), Citrus (*Poncirus trifoliate*) (Xu et al., 2021), Onion (*Allium cepa* L.) (Li et al., 2023), Faba bean (*Vicia faba* L.) (Zhao et al., 2023), Guava (*Psidium guajava* L.) (Maan et al., 2023) and Sesame (*Sesamum indicum*) (Mei et al., 2017). However, there has been limited research on the application of SLAF-seq technology in constructing genetic maps for maize recombinant inbred lines (RIL) mapping populations. In this study, we utilized SLAF-seq technology to develop 10,112 polymorphic markers, enabling the construction of a high-density genetic map for the RIL-F_2:8_ population in maize. Specifically, the three objectives of this study were to: (1) construct a high-density genetic map for the RIL-F_2:8_ population using SLAF-based methods, (2) elucidate the genetic architecture of thermotolerant traits during flowering and identify candidate genes responsible for thermotolerance, and (3) facilitate molecular marker-assisted breeding to expedite the development of new thermotolerant maize varieties.

## Materials and methods

### Plant materials

The 254 RIL-F_2:8_ population were obtained by single seed decent (SSD) from the F_2_ population of a cross between parental inbred lines B73 and Abe2. The representative inbred line B73 exhibited a leaf scorching (thermosensitive) phenotype when subjected to high temperature stress above 35°C during the flowering stage in a field environment. And the native waxy maize inbred line Abe2 in northwestern China exhibited a leaf scorch-free (thermotolerant) phenotype under the same conditions. The 254 RIL-F_2:8_ population and their parents used to construct the high-density genetic map were planted in Ledong County, Hainan, China (18°45N, 109°10E) in 2016.

### Thermotolerant experimental design

Research by Frey et al. showed that leaf scorching trait was one of the important thermosensitive phenotypes under high temperature stress at flowering in maize (Frey et al., 2016). The leaf scorching phenotype in this study was divided into three categories: Leaf Scorching damage (LS), Leaf Scorching Degree (LSD), and Leaf Scorching Ratio (LSR). Phenotypic data of the three types for the LS trait were collected by visual method: extreme leaf scorching damage (phenotypes of leaf scorching are consistent with B73), leaf scorch-free damage (phenotypes of leaf scorching are consistent with Abe2.), and intermediate type. LSD represented the proportion of the leaf scorching damage area in the whole leaves, indicating the degree of leaf scorching damage. It is indicated by the Roman number 1 (no leaf scorching damage) to 9 (extreme leaf scorching damage), with a total of 9 indication levels. LSR referred to the ratio of the number of scorching leaves to the total number of leaves, expressed as 0 to 100%. The broad-sense heritability of leaf scorching traits was calculated according to the following formula:


H2 = σg2/(σg2 + σe2)

where σ_g_^2^ represented the variance of genetic effects, and σ_e_^2^ represents the variance of environmental effects (Yang et al., 2019). A basic statistical analysis was implemented by the SPSS16.0 software with default parameters (SPSS Inc., Chicago, IL, USA) (Zhu et al., 2019).

In view of the continuous high temperature weather conditions in Hefei over the past years, we planted the 254 RIL-F_2:8_ population and their parents for three biological replicates per year in Dayang experimental farm of Anhui Agricultural University (31°49N, 117 °13E) with interval contrast design (ICD) in early June 2017 and 2018 (Zeng et al., 2020). During the maize jointing to flowering stage, they were subjected to continuous high temperature stress, as shown in **Supplementary Figure 1**. In fact, 24 and 29 days of high temperatures above 35°C in 2017 and 2018, respectively.

### DNA extraction, SLAF library construction and high-throughput sequencing

When the 254 RIL-F_2:8_ population and parental inbred lines grew to the V6 period (6 visible leaves), fresh green leaves were removed and stored in the dry ice. Extraction of total genomic DNA for experimental samples using a modified Cetyltrimethyl ammonium bromide (CTAB) method (Murray and Thompson, 1980). An improved SLAF-seq strategy was utilized in our experiment. In this experiment, *Oryza sativa* L *Geng*/*japonica* was used as the control, and the evaluation of the control data monitored whether the experimental process was normal and the effectiveness of the digestion protocol was determined. First, the pre-designed scheme of SLAF is selected using the training data. B73_RefGen_V4 reference genome of maize was used to simulate the number of markers produced by different enzymes, designing marker identification experiments. Next, SLAF library construction was conducted according to a pre-designed scheme. For the 254 RIL population, two enzymes (HaeIII and Hpy166II, New England Biolabs, NEB, USA) were used to digest the genomic DNA at 37°C. A single nucleotide (A) overhang was added subsequently to the digested fragments using Klenow Fragment (3´→ 5´) and dATP (New England Biolabs, NEB, USA). Duplex tag-labeled sequencing adapters (PAGE-purified, Life Technologies, USA) were then ligated to the A-tailed fragments using T4 DNA ligase. Polymerase chain reaction (PCR) was performed using diluted restriction-ligation DNA samples, dNTP, Q5^®^ High-Fidelity DNA Polymerase and PCR primers (Forward primer: 5’-AATGATACGGCGACCACCGA-3’, reverse primer: 5’-CAAGCAGAAGACGGCATACG-3’) (PAGE-purified, Life Technologies). PCR products were then purified using Agencourt AMPure XP beads (Beckman Coulter, High Wycombe, UK) and pooled. Pooled samples were separated by 2% agarose gel electrophoresis. Fragments ranging from 414 to 464 base pairs (with indexes and adaptors) in size were excised and purified using a QIAquick gel extraction kit (Qiagen, Hilden, Germany). Gel-purified products were then diluted. And pair-end sequencing was performed on an Illumina platform system (Illumina, Inc; San Diego, CA, USA) according to the manufacturer’s recommendations.

### Sequence data grouping and genotyping

The SLAF marker identification and genotyping were performed according to procedures described by Sun et al. (Sun et al., 2013). Briefly, low-quality reads were filtered out and then raw reads were sorted to each subsequence according to duplex barcode sequences. After the barcodes and the terminal 5-bp positions were trimmed from each high-quality reads, clean reads from the same sample were mapped onto the maize genome sequence using SOAP software (Li et al., 2008). The reference genome information is based on version B73_RefGen_v4. Sequences mapping to the same position were defined as one SLAF locus (Zhang et al., 2015). Single nucleotide polymorphism (SNP) loci of each SLAF locus were then detected between parents, and SLAFs with more than 3 SNPs were filtered out firstly. In order to obtain high-quality SLAF markers for genetic map construction, first, the average sequence depth should be >21X for parents, while for each offspring the reads with sequence depth >8X were used to define alleles. Second, markers with a data missing rate of more than 50% were filtered. Third, Chi-square test was used to detect segregation distortion. In the process of map construction, markers with significant partial separation (P<0.05) were treated as an auxiliary marker. SLAFs with two to four alleles were identified as polymorphic and considered potential markers. All polymorphism SLAFs loci were genotyped with consistency in the parental and offspring SNP loci. The marker code of the polymorphic SLAFs were analysed according to the RIL population type, which consisted of one segregation types (aa×bb).

### Linkage map construction

SLAF loci were partitioned primarily into linkage groups (LGs) based on their locations on B73_RefGen_V4 reference genome of maize. Next, the modified logarithm of odds (MLOD) scores between SLAF markers were calculated to further confirm the robustness of markers for each LGs. Markers with MLOD scores< 5 were filtered prior to ordering. To ensure efficient construction of the high-density and high-quality map, a newly developed HighMap strategy was utilized to order the SLAF markers and correct genotyping errors within LGs (Liu et al., 2014). Firstly, recombinant frequencies and LOD scores were calculated by two-point analysis, which were applied to infer linkage phases. Then, enhanced Gibbs sampling, spatial sampling and simulated annealing algorithms were combined to conduct an iterative process of marker ordering (Jansen et al., 2001). Briefly, in the first stage of the ordering procedure, SLAF markers were selected using spatial sampling. One marker was taken randomly in a priority order of test cross, and markers with a recombination frequency smaller than a given sampling are excluded from the marker set. Subsequently, simulated annealing was applied to searching for the best map order. Summation of adjacent recombination fractions was calculated as illustrated by (Liu et al., 2014). The annealing system continued until, in a number of successive steps, the newly generated map order is rejected. Blocked Gibbs sampling was employed to estimate multipoint recombination frequencies of the parents after the optimal map order of sample markers were obtained. The updated recombination frequencies were used to integrate the two parental maps, which optimize the map order in the next cycle of simulated annealing. Once a stable map order was obtained after 3-4 cycles, we turned to the next map construction round. A subset of currently unmapped markers was selected and added to the previous sample with decreased sample radius. The mapping algorithm repeats until all the markers were mapped appropriately. The error correction strategy of SMOOTH was then conducted according to parental contribution of genotypes (Van Os et al., 2005), and a k-nearest neighbor algorithm was applied to impute missing genotypes (Huang et al., 2021). Skewed markers were then added into this map by applying a multipoint method of maximum likelihood. Map distances were estimated using the Kosambi mapping function (Kosambi, 2016).

### QTL analysis of thermotolerant traits

The QTL mapping of thermotolerant traits was performed by R/qtl software (Broman et al., 2003) for composite interval mapping (CIM) analysis, and the logarithm of odds (LOD) significance threshold levels was determined by 1000-permutation test (P<0.05). The confidence interval for each QTL was defined using a 2-LOD support interval (Li et al., 2016). The mapping interval of each QTL was determined by the the peak of the LOD and its surrounding value (≥ 2). The software used for drawing the map were origin 7.0 and HighMap. We estimated additive effects and the phenotypic variance explained by individual QTL by the coefficient of determination (R^2^). Positive additive effects indicated favorable alleles derived from Abe2, while negative additive effects indicated favorable alleles from B73.

### Candidate gene analysis, RNA extraction and relative quantitative analysis

After the major QTLs for LS trait were identified, the genetic effect analysis of each QTL locus was carried out to determine the QTL locus with the greatest effect. Combined with previously published QTL-seq results to identify final candidate genes controlling thermotolerant traits (Zeng et al., 2020). The qPCR primers of candidate genes were designed to carry out relative quantitative analysis, with ubiquitin as an internal reference gene. (**Supplementary Table 1**). The parental lines Abe2 and B73 were germinated and grown in a growth chamber at 28°C until the stage of visible 4-leaf growth. Subsequently, a high-temperature stress experiment was conducted in a plant light incubator at a temperature of 42°C and a humidity of 65%. 2×Spark Taq PCR Master Mix (with dye) (Shandong Sparkjade Biotechnology Co., Ltd.) is used for PCR amplification. TRIzol kit (TIANGEN, W9330) was used to extract RNA from fresh leaf, and SPARKscript II RT kit (With gDNA Eraser) (Shandong Sparkjade Biotechnology Co., Ltd.) was used to reverse message RNA into cDNA, which was diluted by five times and amplified by 2×SYBR Green qPCR Mix (With ROX) (SparkJade, AH0104-B).

## Results

### SLAF sequencing and genotyping of the RIL-F_2:8_ population

To develop high-density polymorphic molecular markers, we performed high-throughput SLAF sequencing of the 254 RIL-F_2:8_ population. This resulted in a total of 1,366.12 million reads and 273.03 Gb raw data, with an average Q30 percentage of 93.03% and an average GC percentage of 42.79% (**Supplementary Figure 1A, B** and **Table 1**). For the paternal inbred line (Abe2), a total of 684,620 SLAF markers were developed with an average sequencing depth of 18.48×. The maternal inbred line (B73) produced 955,168 SLAF markers with an average sequencing depth of 23.95×. In addition, 420,299 SLAF markers were developed for the RIL-F_2:8_ population, with an average sequencing depth of 8.05× (**Supplementary Table 2**).

**Table 1:** High-throughput SLAF sequencing data for the RIL-F_2:8_ population in maize.

Sample	Total Read	Total Bases (bp)	Q30 percentage (%)	GC percentage (%)
**Abe2 (P)**	25,152,840	5,029,378,190	92.64	44.11
**B73 (M)**	29,848,184	5,968,717,354	92.55	44.23
**offspring**	5,023,429	1,003,972,479	93.03	42.78
**Total**	1,366,116,164	273,034,912,606	93.03	42.79

Q30 percentage (%): Percentage of bases with a sequencing quality value ≥30;

GC percentage (%): Guanine (G) and cytosine (C) as a percentage of total bases in the sequencing results;

Offspring: Average of the sequencing data of the offspring;

P: paternal inbred line;

M: maternal inbred line.

**Figure 1:**
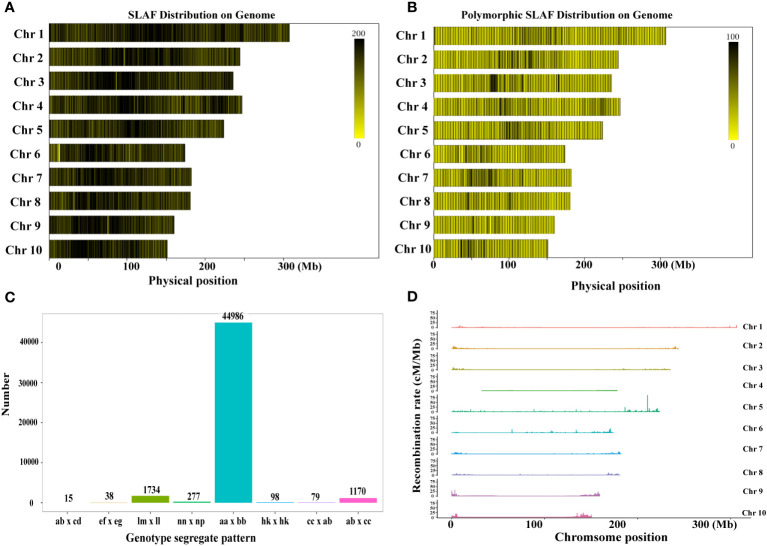
Development of SLAF markers for the RIL-F_2:8_ population in maize. **(A)** The distribution of SLAF markers on each chromosome. **(B)** The distribution of the polymorphic SLAF markers on each chromosome. The more SLAF markers, the darker the color, and the fewer SLAF markers, the lighter the color. **(C)** Genotype distribution of polymorphic SLAF markers. The x-axis indicates eight segregation patterns of polymorphic SLAF markers, the y-axis indicates the number of markers. **(D)** Recombination rate of polymorphic SLAF markers on each chromosome.

The SLAF markers were aligned to the maize reference genome using BWA software (Van Os et al., 2005), and the count of SLAF markers and polymorphic SLAF markers on each linkage group was determined. The length of SLAF markers ranged from 414 bp to 464 bp (**Supplementary Figure 1C**). Polymorphism analysis was conducted based on the variation in allele number and marker sequence, resulting in three types of SLAF markers: Polymorphic, Non-Polymorphic, and Repetitive SLAF (**Figure 1**; **Supplementary Table 3**, **4**). A total of 589,770 SLAF markers developed for all samples were used for SLAF polymorphism marker analysis (**Supplementary Table 3**). Among them, 108,709 were identified as polymorphic SLAF markers, with a polymorphism ratio of 18.43% (**Supplementary Table 3**). The distribution of SLAF markers and polymorphic SLAF markers across each linkage group was visualized (**Figures 1A, B**; **Supplementary Table 4**), indicating an even distribution of SLAF markers on each linkage group.

To facilitate further genetic analysis, the polymorphic markers were encoded following the general 2-allelic coding rules of genetics (**Supplementary Table 5**). After filtering out the SLAF markers with parental information, a total of 108,709 polymorphic SLAF markers were genotyped and classified into eight segregation patterns (**Figure 1C**). Additionally, we calculated the recombination values between all polymorphic SLAF markers and observed fewer recombination hotspots on each chromosome (**Figure 1D**). Based on the genetic characteristics of the RIL-F_2:8_ population, the aa x bb type polymorphic markers were selected as valid markers. Consequently, 48,397 markers belonged to the valid marker category of the aa x bb type, resulting in an effective polymorphism rate of 8.21% (**Supplementary Table 3**).

### Construction of a high-density genetic map based on the polymorphic SLAF markers

To ensure the quality of the genetic map, the polymorphic SLAF markers underwent several filtering steps. First, markers were filtered based on parental sequencing depth, ensuring it was above 6X. Then, markers with less than 5 SNPs were excluded. Completeness filtering was applied, requiring the genotype to cover at least 50% of all progeny individuals. Additionally, markers showing partial separation were filtered out. After this process, 10,724 polymorphic SLAF markers remained for genetic map construction. Subsequently, markers with MLOD values lower than 5 when compared with other SLAF markers were further filtered (Huang et al., 2011), resulting in a final count of 10,112 polymorphic markers used for constructing the genetic map (**Table 2**).

**Table 2:** Basic information of a high-density genetic map based on the 10,112 polymorphic SLAF markers.

LG	SLAF Markers	Total Distance (cM)	Average Distance (cM)	Gaps≤5 (cM)	Max Gap (cM)
**1**	1,204	191.22	0.16	100.00%	2.67
**2**	962	151.54	0.16	100.00%	2.67
**3**	935	166.06	0.18	100.00%	2.45
**4**	713	105.06	0.25	100.00%	3.36
**5**	2,121	187.12	0.09	100.00%	4.93
**6**	1,319	154.09	0.12	100.00%	4.46
**7**	1,249	160.30	0.13	99.92%	5.9
**8**	1,151	146.36	0.13	100.00%	2.67
**9**	240	103.94	0.73	98.74%	10.9
**10**	218	110.20	0.68	100.00%	4.21
**Total**	10,112	1,475.88	0.16	99.87%	10.9

**Figure 2:**
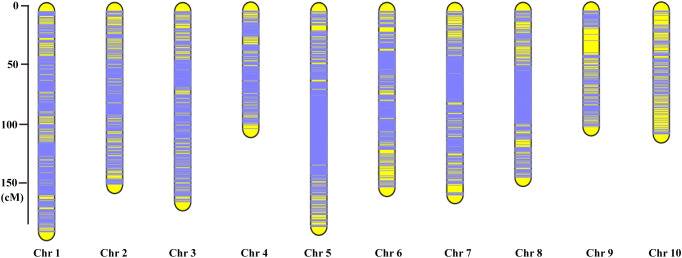
The high-density genetic map constructed based on the 10,112 valid polymorphic SLAF markers for maize F_2-8_ RIL population. The x-axis represents the linkage group number, the y-axis indicates the genetic distance (cM) within each linkage group.

Each chromosome served as a linkage group, and HighMap software (Li et al., 2018) was utilized to analyze the linear array of SLAF markers. Genetic distances between adjacent markers were estimated, culminating in the construction of a genetic map with a total map distance of 1,475.88 cM (**Table 2**; **Figure 2**). The high-density genetic map had an average genetic distance of 0.16 cM, with 98.77% of the gaps being less than 5 cM and distributed nearly evenly across each linkage group (**Table 2**). Among the 10 chromosomes, chromosome 9 exhibited the largest gap of 10.9 cM due to an insufficient number of polymorphic markers, resulting in an average genetic distance of 0.73 cM, the highest among all linkage group maps. Conversely, chromosome 5 boasted the highest number of markers, with 2,121 polymorphic SLAF markers and the smallest average genetic distance of 0.09 cM among all linkage group maps (**Table 2**).

### Comprehensive evaluation and high-quality construction of a genetic map using SLAF-seq technology

In order to assess the quality of the genetic map, a comprehensive evaluation of various aspects was conducted to ensure its accuracy and reliability. These aspects include analyzing the collinearity between the map position of SLAF markers and their physical location on the genome (**Supplementary Table 6**), examining the sequencing depth of the markers (**Supplementary Table 7**), analyzing SNP markers within each linkage group (**Table 3**), assessing segregation distortion (**Supplementary Table 9**), evaluating the integrity of markers across all individuals (**Supplementary Figure 3**), conducting monomer analysis for individual genotypes (**Supplementary Figure 4**), and studying the recombination relationship between SLAF markers and adjacent ones (**Supplementary Figure 4**). Each aspect provided valuable insights into the quality and reliability of the genetic map.

**Table 3:** Basic information of SNP loci of 10 linkage groups (LG).

LG	SNP Number	Trv	Tri	Trv/Tri
**1**	2,351	670	1,681	0.4
**2**	1,795	524	1,271	0.41
**3**	1,738	499	1,239	0.4
**4**	1,418	397	1,021	0.39
**5**	3,611	979	2,632	0.37
**6**	2,439	681	1,758	0.39
**7**	2,206	613	1,593	0.38
**8**	1,908	496	1,412	0.35
**9**	454	144	310	0.46
**10**	389	106	283	0.37
**Total**	18,309	5,109	13,200	0.39

Trv: SNP transversion.

Tri: SNP conversion.

The quality of the genetic map has a direct impact on subsequent QTL mapping and genetic analysis. Upon evaluating the seven key aspects mentioned above, it is evident that this experiment yielded a high-quality, high-density genetic map. The map order of SLAF markers aligns consistently with the physical genome location (**Supplementary Table 6**), with each molecular marker exhibiting an average sequencing depth exceeding 10X (**Supplementary Table 7**). Furthermore, the genotype integrity of each individual reached 99.83% (**Supplementary Figure 3**), and the allelic origins for larger genetic segments in each individual demonstrated high consistency (**Supplementary Figure 4**). In summary, we utilized SLAF-seq technology to develop 10,112 polymorphic molecular markers, resulting in the construction of a high-quality, high-density genetic map.

### Understanding the impact of high temperature stress on maize leaf scorching phenotype

Frey et al. proposed that leaf scorching is a crucial thermosensitive phenotype during the reproductive stage of maize under high temperature stress (Frey et al., 2016). A survey of daily temperatures throughout the maize growth period revealed that the parental inbred lines and RIL-F_2:8_ population encountered temperatures exceeding 35°C for up to 17 days in 2017 and 19 days in 2018 (**Supplementary Figure 1**). Following prolonged exposure to high temperatures, the parent B73 displayed noticeable leaf scorching damage, whereas the parent Abe2 remained unscathed by leaf scorching (Zeng et al., 2020). The RIL-F_2:8_ population exhibits a remarkably diverse range of phenotypic variations, characterized by various levels of thermosensitive phenotypes under prolonged exposure to high temperature stress. (**Supplementary Figure 5**, **6**; **Supplementary Table 8**). Correlation coefficients between the three indicators of the leaf scorching trait were calculated as 0.146 (between LS and LSR), 0.144 (between LS and LSD), and 0.875 (between LSR and LSD) (**Supplementary Figure 6**). Notably, a highly significant positive correlation between LSR and LSD was observed, suggesting these two indicators could serve as reliable references. The broad-sense heritability values for the three indicators were 0.73 (LS), 0.38 (LSD), and 0.60 (LSR), respectively (**Supplementary Table 10**). Despite the strong correlation between LSR and LSD, their heritability differed significantly, with LS exhibiting the highest heritability and LSD the lowest. Overall, this research sheds light on the complex relationship between high temperature stress and leaf scorching in maize, providing valuable information for understanding and potentially improving the plant’s resilience to such environmental challenges.

### Genetic architecture feature of thermotolerance traits in the RIL-F_2:8_ population

**Figure 3:**
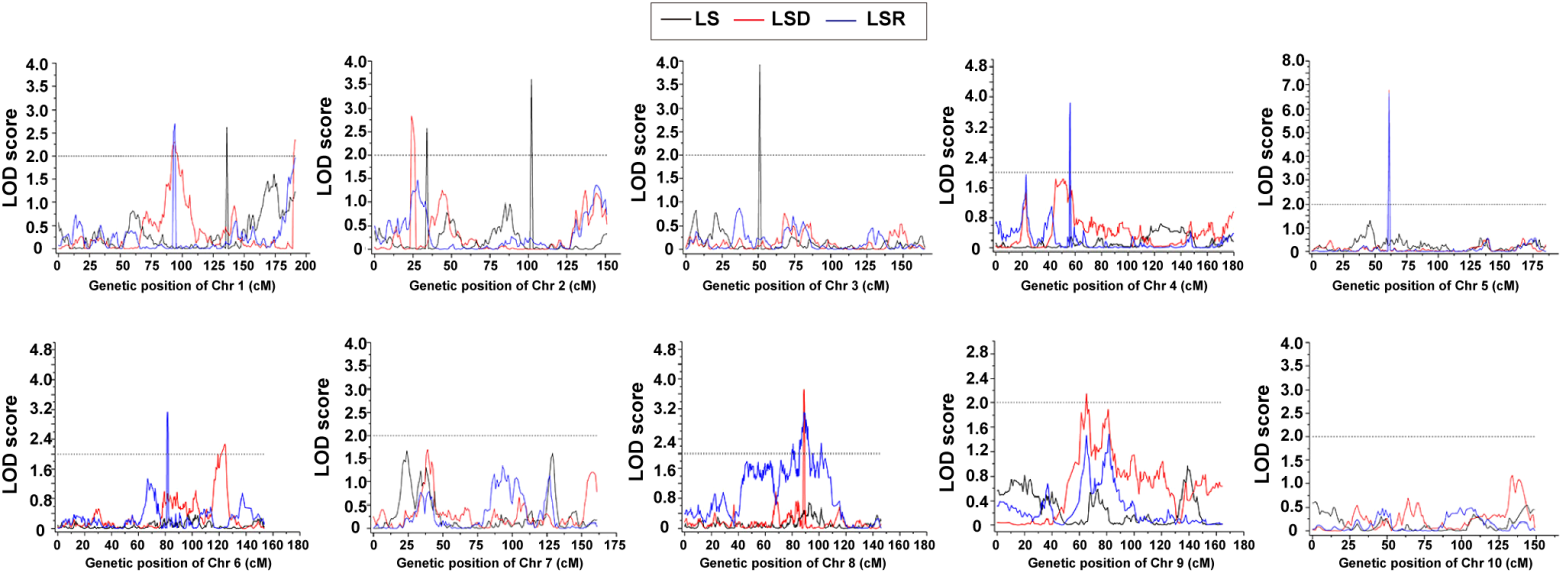
The QTLs of thermotolerance traits identified in the RIL-F_2:8_ population using a high-density genetic map. Thermotolerance trait was divided into three types: LS (Leaf Scorching damage, Black lines), LSD (Leaf Scorching Degree, Red lines) and LSR (Leaf Scorching Ratio, Blue lines). The x-axis indicated genetic position (cM), the y-axis indicated LOD score.

Based on the high-density genetic map and phenotypic characterization, we performed QTL analysis of thermotolerance traits using composite interval mapping (CIM) method of R/qtl software. A total of 16 QTLs were identified (**Table 4**, **Supplementary Table 11**; **Figure 3**), including 4 QTLs belonging to LS, and 6 QTLs each for LSD and LSR, respectively. The four LS QTLs were distributed on chromosomes 1 (*qLS1*), 2 (*qLS2.1*, *qLS2.2*) and 3 (*qLS3*), explaining a phenotypic variation range between 2.83% and 6.58% (**Table 4**). The six LSD QTLs were located on chromosome 1, 2, 5, 6, 8 and 9 respectively, explaining a phenotypic variation range between 2.13% and 9.11%. The genetic architecture of LSD featured a large-effect QTL along with many small-effect QTLs (**Table 4**). The six LSR QTLs were located on chromosomes 1, 4, 5, 6, and 8, respectively, collectively explaining 25.98% of the phenotypic variation. Further genetic architecture information for thermotolerance traits could be found in (**Table 4**, **Figure 3**; **Supplementary Figure 7**). Among the 16 QTLs, the favorable allele loci of 11 QTLs were from the parental inbred line Abe2, while the favorable allele loci of the remaining QTLs were from the maternal inbred line B73 (**Supplementary Figure 7**). The localization results and chromosomal distribution of the 16 QTL loci associated with thermosensitive phenotype traits are presented in **Supplementary Figure 8**. Chromosome 1, 2, and 8 each harbor three QTL loci, while chromosome 5 and 6 contain two QTL loci each. Lastly, chromosome 3 and 4 each have one QTL locus. Most of the QTL loci span large genomic regions.

**Table 4:** The QTLs of thermotolerance traits identified in the RIL-F_2:8_ population using a high-density genetic map.

Traits	no. of QTLs	Variation explained by each QTL (%)	Variation explained by all QTL (%)	Genetic architecture feature
**LS**	4	2.83-6.58	19.73	many small-effect QTLs
**LSD**	6	2.13-9.11	27.98	a large-effect QTL plus many small-effect QTLs
**LSR**	6	2.39-6.89	25.98	many small-effect QTLs

### Identification and characterization of candidate genes influencing thermotolerance traits by combining high-density genetic map and QTL-seq strategy

**Figure 4:**
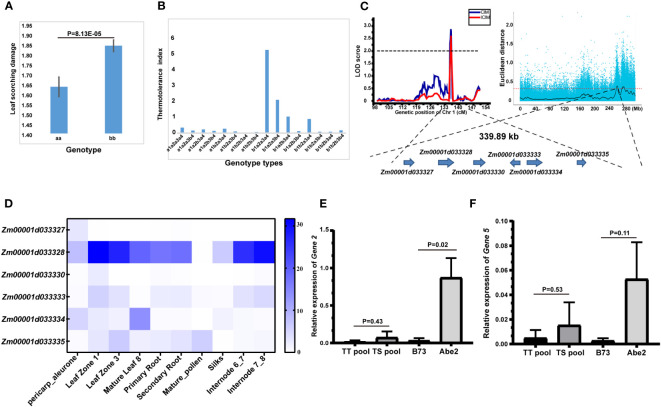
Identification of candidate genes for the thermotolerance traits by combining high-density genetic map and QTL-seq strategy. **(A)** Allele significance analysis of *qLS1* controlling the thermotolerance traits. **(B)** Genetic effects of 4 QTLs of leaf scorching damage (LS) controlling the thermotolerance traits. a1b1 represented the allele of *qLS2.2*. a2b2 represented the allele of *qLS2.1*. a3b3 represented the allele of *qLS3*. a4b4 represented the allele of *qLS1*. **(C)** Candidate genomic region on chromosome 1 controlling the thermotolerance traits by combining linkage genetic analysis and QTL-seq strategy. **(D)** Tissue expression patterns of six candidate genes. **(E)** Relative expression of candidate *Gene 2* under high temperature stress. **(F)** Relative expression of candidate *Gene 5* under high temperature stress. The significance between different treatment groups was examined using the t-test method.

Among the 16 identified QTLs associated with thermotolerance traits, the *qLS1* allele displayed a notable difference of 8.13E-05 (**Figure 4A**), pointing to its significance as a key candidate locus. Additionally, we conducted an analysis of the genetic effects related to four QTL loci governing the LS trait, with the outcomes depicted in **Figure 4B**. Notably, when the allele of *qLS2.2* was denoted as a1, and the alleles of the other three loci were a1 or b1, the thermotolerance index of the RIL-F_2:8_ individuals generally exhibited lower levels, highlighting the pivotal role of the *qLS2.2* locus in controlling the LS trait. Furthermore, the majority of RIL-F_2:8_ individuals displayed leaf scorching phenotypes when possessing the a1 allele of the *qLS2.2* locus. Conversely, when the allele of *qLS2.2* was b1, and alleles of *qLS2.1* and *qLS3* were a2a3, the RIL-F_2:8_ individuals demonstrated higher thermotolerance indices, particularly when the allele of *qLS1* was a4, signifying its crucial role in regulating the LS trait (**Figure 4B**). Notably, the allele of *qLS1* was a4, the RIL-F_2:8_ individuals could exhibit strong thermotolerance. Furthermore, in conjunction with preceding QTL-seq results from our laboratory (Zeng et al., 2020), a co-localization on chromosome 1 was discovered, encompassing only six genes governing the thermotolerance trait (**Figure 4C**).

The annotation details for the six candidate genes are provided in **Table 5**. *Gene 1* is identified as a 2-oxoglutarate (2OG) and Fe (II)-dependent gene, potentially implicated in redox processes. *Gene 2* is characterized as an N-acetyl-gamma-glutamyl-phosphate reductase, also likely involved in redox processes. *Gene 3* belongs to the Cyclin class and may play a role in regulating the cell cycle. *Gene 4* is classified as an ARM repeat superfamily protein with no associated functional annotation. *Gene 5* is described as a Calcium-transporting ATPase 2 plasma membrane-type, possibly involved in ion transport processes. Lastly, *Gene 6* is identified as a LOB domain-containing protein 28, potentially involved in ATP hydrolysis-coupled cation transmembrane transport processes.

**Table 5:** The annotation information of the candidate genes controlling the thermotolerance trait.

Gene	Gene ID_AGPv4	Chr	Start (bp)	Stop (bp)	Gene annotation	Related phenotype in other plants	Homologs
***Gene 1***	*Zm00001d033327*	1	259129101	259130297	2-oxoglutarate (2OG) and Fe(II)-dependent oxygenase superfamily protein	no	AT3G19010.1
***Gene 2***	*Zm00001d033328 *	1	259165238	259169626	N-acetyl-gamma-glutamyl-phosphate reductase	no	AT2G19940.1
***Gene 3***	*Zm00001d033330*	1	259214061	259215909	Cyclin-D5-1	no	AT4G37630.1
***Gene 4***	*Zm00001d033333*	1	259324966	259330535	ARM repeat superfamily protein	no	AT1G64960.1
***Gene 5***	*Zm00001d033334*	1	259330315	259335855	Calcium-transporting ATPase 2 plasma membrane-type	no	AT4G37640.1
***Gene 6***	*Zm00001d033335*	1	259429013	259429858	LOB domain-containing protein 28	no	AT5G66870.1

Subsequently, the coding sequence variants of the six candidate genes were analyzed, revealing that only four genes exhibited variations in the coding region (**Supplementary Table 12**). Notably, *gene 2* harbored a sole non-synonymous variant SNP. Analysis of the tissue expression patterns of the six candidate genes indicated robust expression of *gene 2*, particularly in leaves and internodes, while the remaining five genes displayed markedly lower expression levels (**Figure 4D**). Furthermore, assessment of the relative expression of the six candidate genes under high-temperature stress unveiled significant differences in the expression of gene 2 between the parents (P<0.05) (**Figure 4E**), with gene 5 exhibiting low expression without significant variance (**Figure 4F**). Conversely, the expression of the remaining four genes was undetectable due to extremely low expression levels. In summary, based on significant differences in both expression levels and coding region sequences, *gene 2* emerges as the prime candidate gene influencing the thermotolerance trait.

We further analyzed the variations in the promoter region and found a total of 1681 SNP variations within the QTL mapping interval, with 22 SNPs located in the coding sequence (CDS) region, most of which were promoter region variations (**Supplementary Table 13**). Specifically, the promoter region of *Zm00001d033328* exhibited 47 SNP variations along with 41 cis-regulatory elements (**Supplementary Table 14**). These elements play a crucial role in various biological processes such as MeJA-responsiveness, light responsiveness, regulation of flavonoid biosynthetic genes, auxin-responsive elements, gibberellin-responsive elements, drought-inducibility, abscisic acid responsiveness, and MeJA-responsiveness.

## Discussion

Genetic map has been widely developed in plants or animals, which has been shown to be useful for a variety of applications, including gene mapping of important agronomic traits or quantitative trait loci (QTL) mapping, map-based cloning, marker-assisted selection breeding, and genome assembly analysis (Foulongne-Oriol, 2012). The correct selection of mapping populations is a prerequisite for effective separation of the population. According to the different stability, the mapping population can be divided into temporary mapping population and permanent mapping population. The mapping population in this study was a highly homozygous F_2:8_ recombinant inbred line (RIL) population, which belonged to the permanent mapping population. Compared with temporary mapping populations, such as F2 population and backcrossing (BC) population, RIL population had the characteristics of stable inheritance, homozygous genotype and easy to reuse.

Compared with the previous genetic map constructed using SSR molecular markers (Mei et al., 2006; Shi et al., 2022), the high-density genetic map constructed by SLAF sequencing technology in this study have the following characteristics: 1, it belongs to the third generation of molecular markers, which is the most abundant molecular marker at the current level; 2. The distribution of molecular markers on each linkage group is more uniform and denser than SSR; 3. Compared with SSR molecular markers, the mapping interval is narrower. The successful construction of a high-density genetic map is an important basis for subsequent QTL mapping of thermotolerance trait at flowering and other agronomic complex traits in maize. In this study, high-throughput SLAF sequencing technology was used to obtain a high-quality, high-density genetic map with a total length of 1,475.88 cM, and 10,112 SLAF markers were uniformly covered on 10 linkage groups (**Table 2**; **Figure 2**). We evaluated the quality of the high-density genetic map by seven aspects of quality assessment (**Supplementary Figures 2–4**; **Figure 1**; **Table 3**; **Supplementary Tables 2**, **6**–**9**), which fully demonstrated that this is a high-quality, high-density genetic map in maize.

Using the constructed high-density genetic map, we analyzed the genetic structure of thermotolerance traits, which could be divided into LS, LSR and LSD. Frey et al. suggested the leaf scorching trait was a phenotype exhibited by high temperature stress during the adult stage of maize, and the trait was divided into nine grades from 1 (weak damage) to 9 (strong damage) (Frey et al., 2016). In this experiment, we also observed that the leaf scorching trait was a phenotype exhibited by high temperature stress during the flowering stage of maize, and the leaf scorching trait was described in more detail (**Supplementary Figure 1**, **6**; **Supplementary Table 10**). Based on the high-density genetic map, a total of 16 QTLs associated with thermotolerance traits were identified and distributed on all chromosomes except the 7 and 10 chromosomes. It was worth mentioning that we used the leaf scorching degree (LSD) indicator consistent with Frey et al. to collect data for leaf scorching from 1 (weak damage) to 9 (strong damage). We found that *qLSD9* was located at 65.17 cM on chromosome 9, which could explain 5.62% of the phenotypic variation, consistent with the mapping results of Frey et al. (Frey et al., 2016). In addition, among the three indicators of thermotolerance traits (LS, LSD, LSR), the broad-sense heritability of the LSD is the lowest, only 0.38, while the broad-sense heritability of the LS could reach to 0.73 (**Supplementary Table 10**). This implied that the LS indicator was more suitable as a measurement index under high temperature stress during the reproductive stage of maize.

In the study, it was observed that only LSR (Leaf Scorching Ratio) and LSD (Leaf Scorching Degree) exhibited a high correlation among the RIL-2:8 population, while LS (Leaf Scorching damage) does not show a strong correlation with the other two indicators. Here are some possible explanations: 1. Genetic factors: The correlation between different traits can be influenced by the underlying genetic factors. It is possible that the genetic basis for LS is different from the genetic basis for LSR and LSD. This could be due to the involvement of different genes or genetic pathways in determining the severity and extent of leaf scorching damage compared to the proportion of scorching leaves. 2. Environmental factors: It is possible that the environmental factors in the specific conditions where the RIL population was evaluated had a stronger influence on LS compared to LSR and LSD. This could lead to a weaker correlation between LS and the other two indicators. 3. Measurement methods: The different measurement methods used for LS, LSR, and LSD could contribute to the variation in their correlations. Visual assessment, as described in your study, might introduce subjectivity and measurement errors. It is possible that the visual method used for LS assessment was less precise or more prone to variation compared to the methods used for LSR and LSD, leading to weaker correlations with LS. 4. Sample size and statistical power: The strength of correlations can be influenced by the sample size and statistical power of the study. It is possible that the RIL population used in your study was not large enough to detect significant correlations between LS and the other indicators. A larger sample size might be needed to uncover potential correlations that were not observed in our study.

In summary, this study was the first to systematically analyze the phenotypic and genetic basis of leaf scorching traits under high temperature stress during the reproductive stage of maize. Through the high-throughput SALF-seq technology, 10,112 polymorphic SLAF markers were developed and a high-quality, high-density genetic map was constructed. This provided an important foundation for the genetic basis of other agronomic complex traits and future marker assisted selection breeding (MAS) in maize. This study also identified six candidate genes associated with thermotolerance traits by combining the high-density genetic maps with the QTL-seq strategy, of which *Zm00001d033328* is the most likely candidate gene for controlling the thermotolerance trait at flowering in maize.

## Data availability statement

The data presented in the study are deposited in the National Center for Biotechnology Information (NCBI) repository, accession number PRJNA824290.

## Author contributions

TW: Formal analysis, Investigation, Visualization, Writing – original draft. XZ: Formal analysis, Investigation, Visualization, Writing – review & editing. JZ: Data curation, Project administration, Writing – review & editing. SZ: Data curation, Formal analysis, Writing – review & editing. MR: Data curation, Investigation, Writing – original draft. WZ: Data curation, Investigation, Writing – original draft. Writing – review & editing.

## Supplementary material

Supplementary Figure 1 Daily maximum temperatures during the maize growing period in 2017-2018.

Supplementary Figure 2 Genomic sequencing quality analysis and SLAF length distribution. **(A)** Distribution of sequencing quality values. The abscissa is the base position of the reads, and the ordinate is the single-base error rate. **(B)** Distribution analysis of base types. The abscissa is the base position of reads, and the ordinate is the proportion of bases; Different colors represent different base types, green represents base A, red represents base C, orange represents base G, blue represents base T, and gray represents base N that cannot be identified in sequencing. **(C)** The 414-464 bp mapped reads are the main SLAF length range.

Supplementary Figure 3 Integrity analysis of SLAF markers in all RIL-F_2:8_ individuals.

Supplementary Figure 4 Monomer analysis (up) for each individual genotype and recombination analysis (down) between the SLAF marker and adjacent SLAF markers. One row in the monomer figure (up) represents a marker, and one column represents one individual originated from the RIL-F_2:8_ population. In recombination figure (down), each row and column are markers that follow the genetic map order. Each small square represents the recombination rate between the two SLAF markers. The change of color from yellow to red to purple represents the recombination rate from small to large.

Supplementary Figure 5 The various levels of thermosensitive phenotypes from RIL-F_2:8_ population under high temperature stress at flowering in maize.

Supplementary Figure 6 Phenotypic variation of thermotolerance traits in the RIL-F_2:8_ population. The figures on the diagonal show the phenotypic distribution of each thermotolerance trait. The values above the diagonal are pairwise correlation coefficients between the thermotolerance traits, and the figures below the diagonal are scatter plots between the thermotolerance traits. *, P< 0.05; **, P< 0.01. LS, Lead Scorching damage, LSD, Leaf Scorching Degree, and LSR, Leaf Scorching Ratio.

Supplementary Figure 7 The R^2^ and additive effect of quantitative trait loci (QTLs) for thermotolerance traits in the RIL-F_2:8_ population. The x-axis indicates each identified QTL, and the y-axis indicates the percentage of phenotypic variance explained by a QTL (up) and additive effects (down) by each QTL.

Supplementary Figure 8 Unveiling the chromosomal distribution of QTLs in Maize under high temperature stress during the flowering stage. The larger the graphic of each QTL locus, the larger the genomic region; the narrower the graphic, the smaller the genomic region.

Supplementary Table 8 The thermosensitive phenotypes from RIL-F2:8 population under high temperature stress at flowering in maize.
