# Supplementary figures and images for: A SLAF-based high-density genetic map construction and genetic architecture of thermotolerant traits in maize (Zea mays L.)
Source: Front Plant Sci. 2024 Feb 7;15:1338086. doi: 10.3389/fpls.2024.1338086 (PMC10880447; doi:10.3389/fpls.2024.1338086)

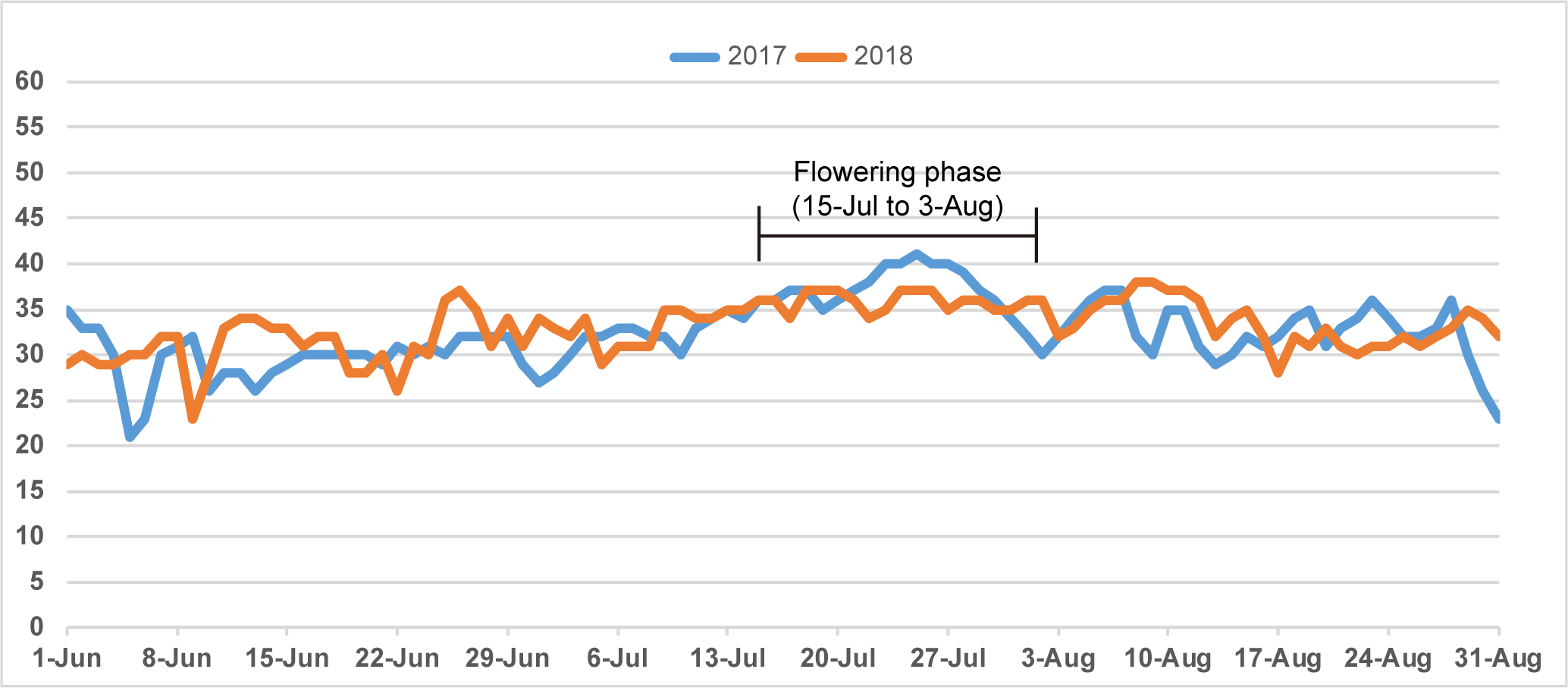

Supplement: Supplementary Figure 1 — Daily maximum temperatures during the maize growing period in 2017-2018. [file Image_1.tif]

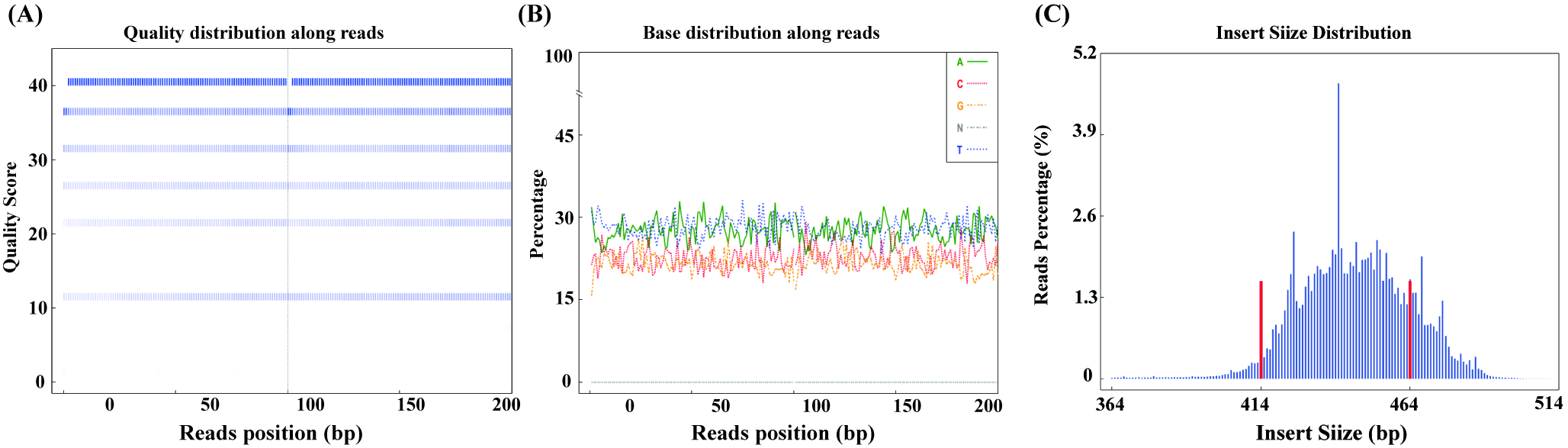

Supplement: Supplementary Figure 2 — Genomic sequencing quality analysis and SLAF length distribution. (A) Distribution of sequencing quality values. The abscissa is the base position of the reads, and the ordinate is the single-base error rate. (B) Distribution analysis of base types. The abscissa is the base position of reads, and the ordinate is the proportion of bases; Different colors represent different base types, green represents base A, red represents base C, orange represents base G, blue represents base T, and gray represents base N that cannot be identified in sequencing. (C) The 414-464 bp mapped reads are the main SLAF length range. [file Image_2.tif]

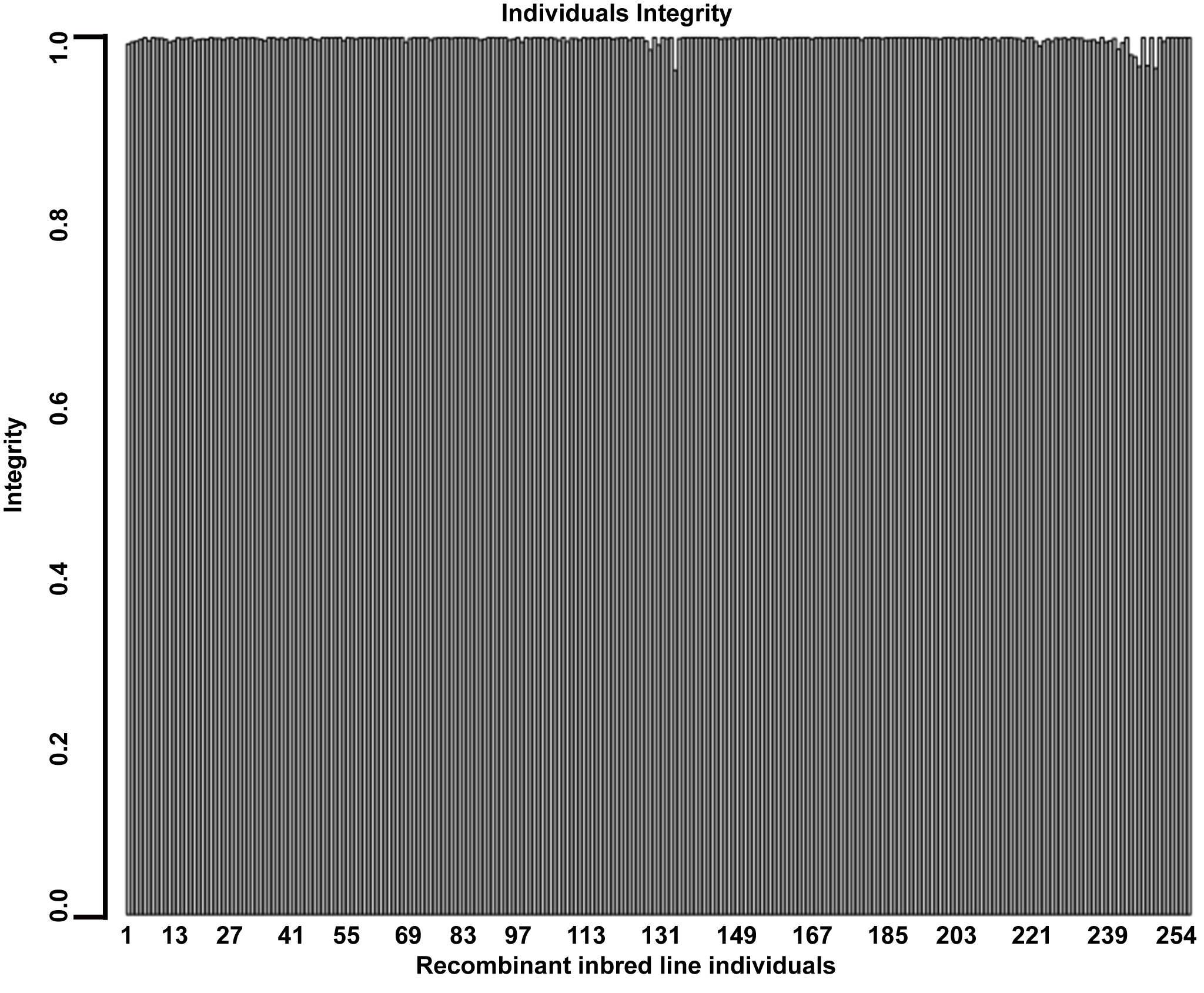

Supplement: Supplementary Figure 3 — Integrity analysis of SLAF markers in all RIL-F2:8 individuals. [file Image_3.tif]

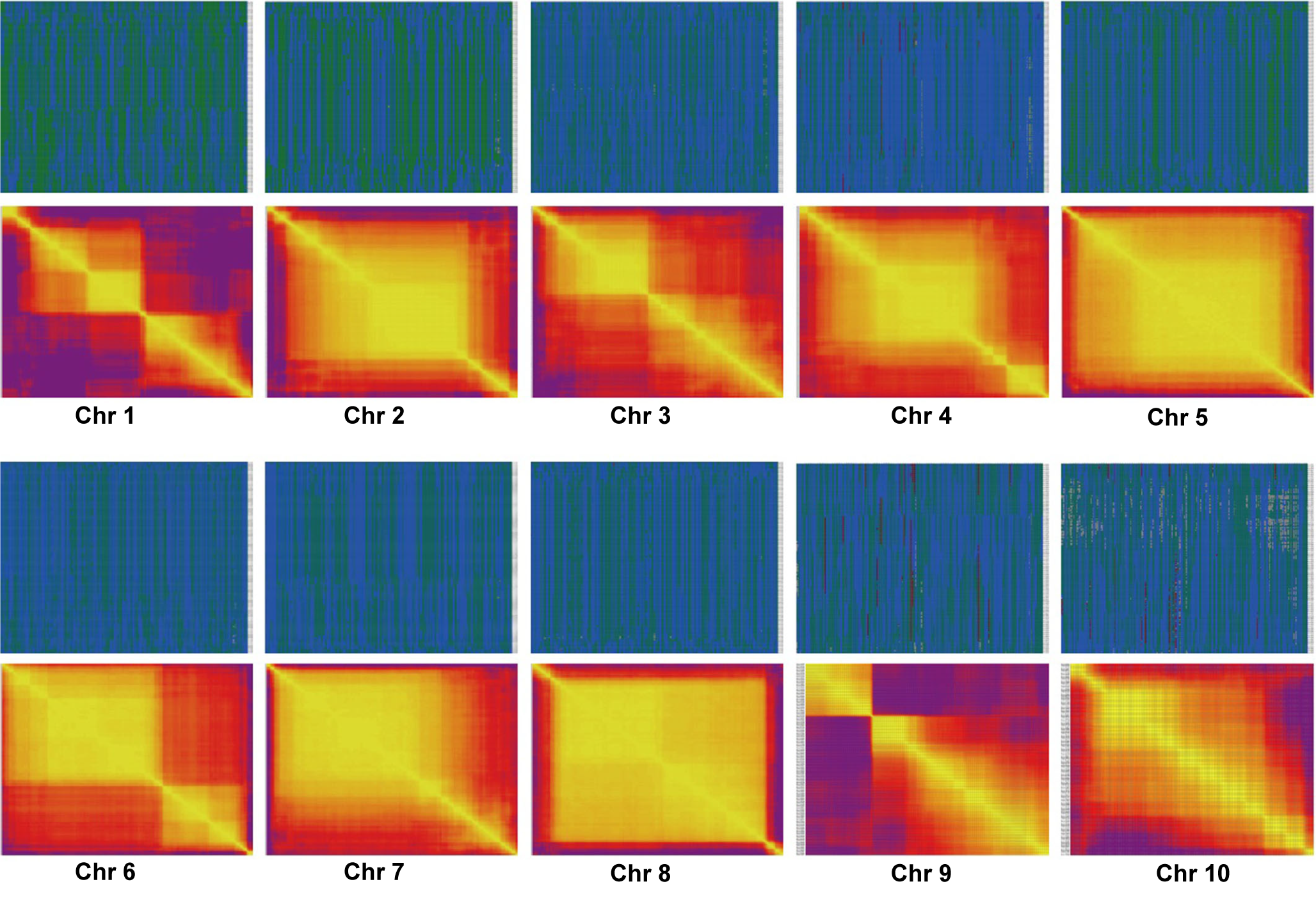

Supplement: Supplementary Figure 4 — Monomer analysis (up) for each individual genotype and recombination analysis (down) between the SLAF marker and adjacent SLAF markers. One row in the monomer figure (up) represents a marker, and one column represents one individual originated from the RIL-F2:8 population. In recombination figure (down), each row and column are markers that follow the genetic map order. Each small square represents the recombination rate between the two SLAF markers. The change of color from yellow to red to purple represents the recombination rate from small to large. [file Image_4.tif]

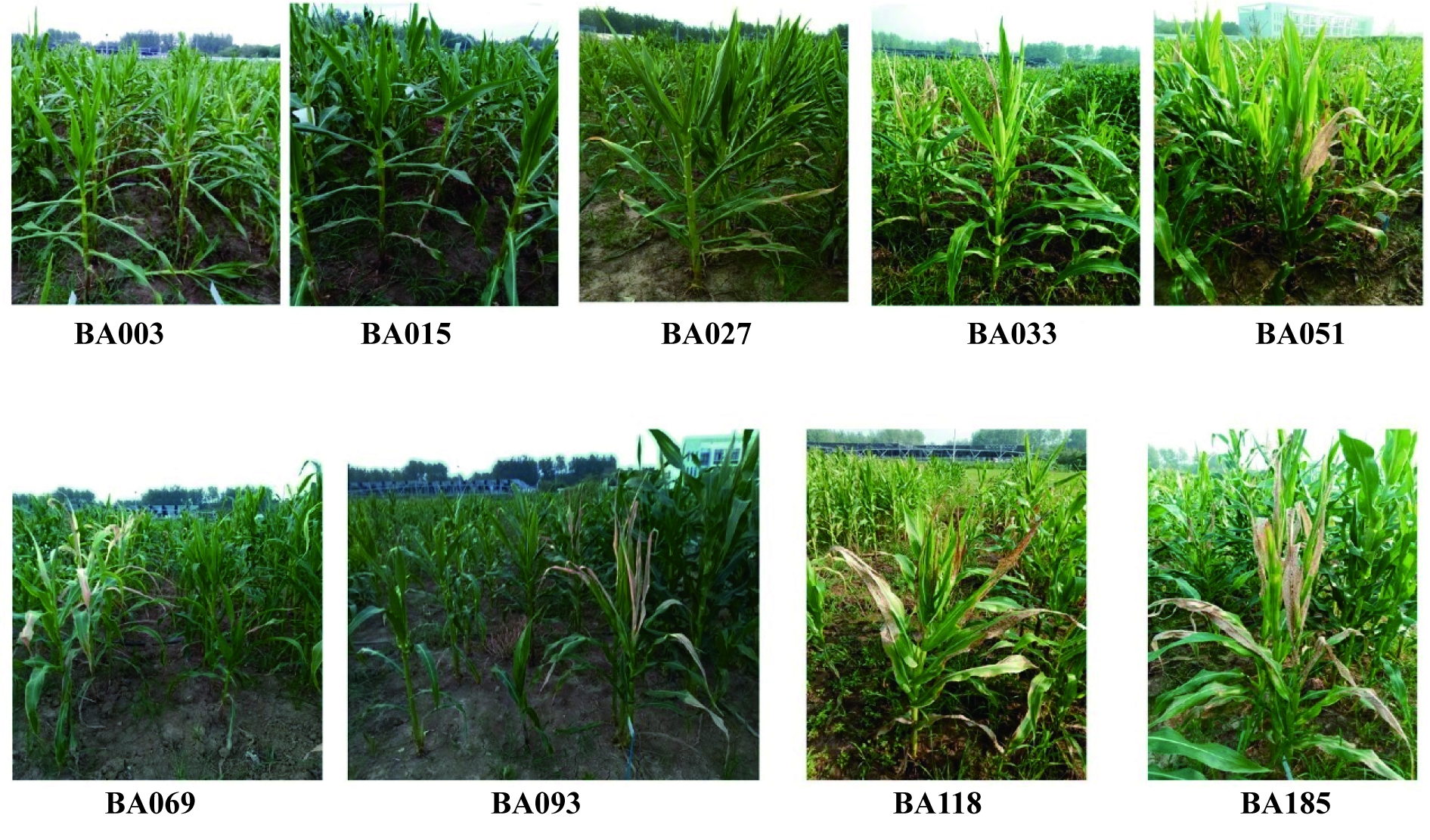

Supplement: Supplementary Figure 5 — The various levels of thermosensitive phenotypes from RIL-F2:8 population under high temperature stress at flowering in maize. [file Image_5.tif]

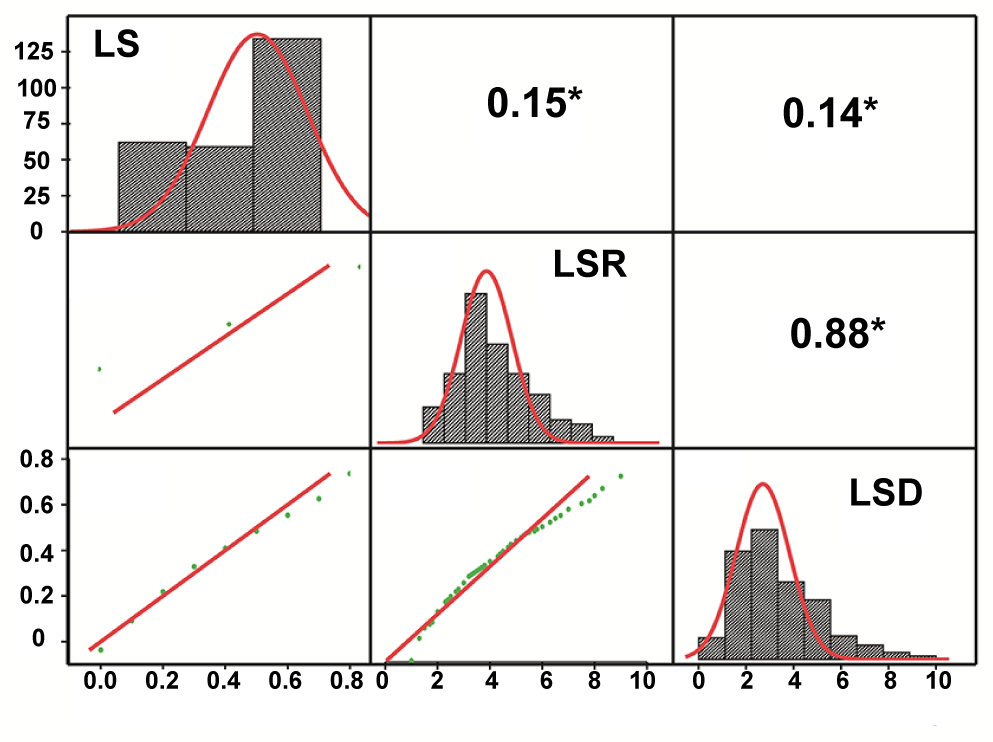

Supplement: Supplementary Figure 6 — Phenotypic variation of thermotolerance traits in the RIL-F2:8 population. The figures on the diagonal show the phenotypic distribution of each thermotolerance trait. The values above the diagonal are pairwise correlation coefficients between the thermotolerance traits, and the figures below the diagonal are scatter plots between the thermotolerance traits. *, P< 0.05; **, P< 0.01. LS, Lead Scorching damage, LSD, Leaf Scorching Degree, and LSR, Leaf Scorching Ratio. [file Image_6.tif]

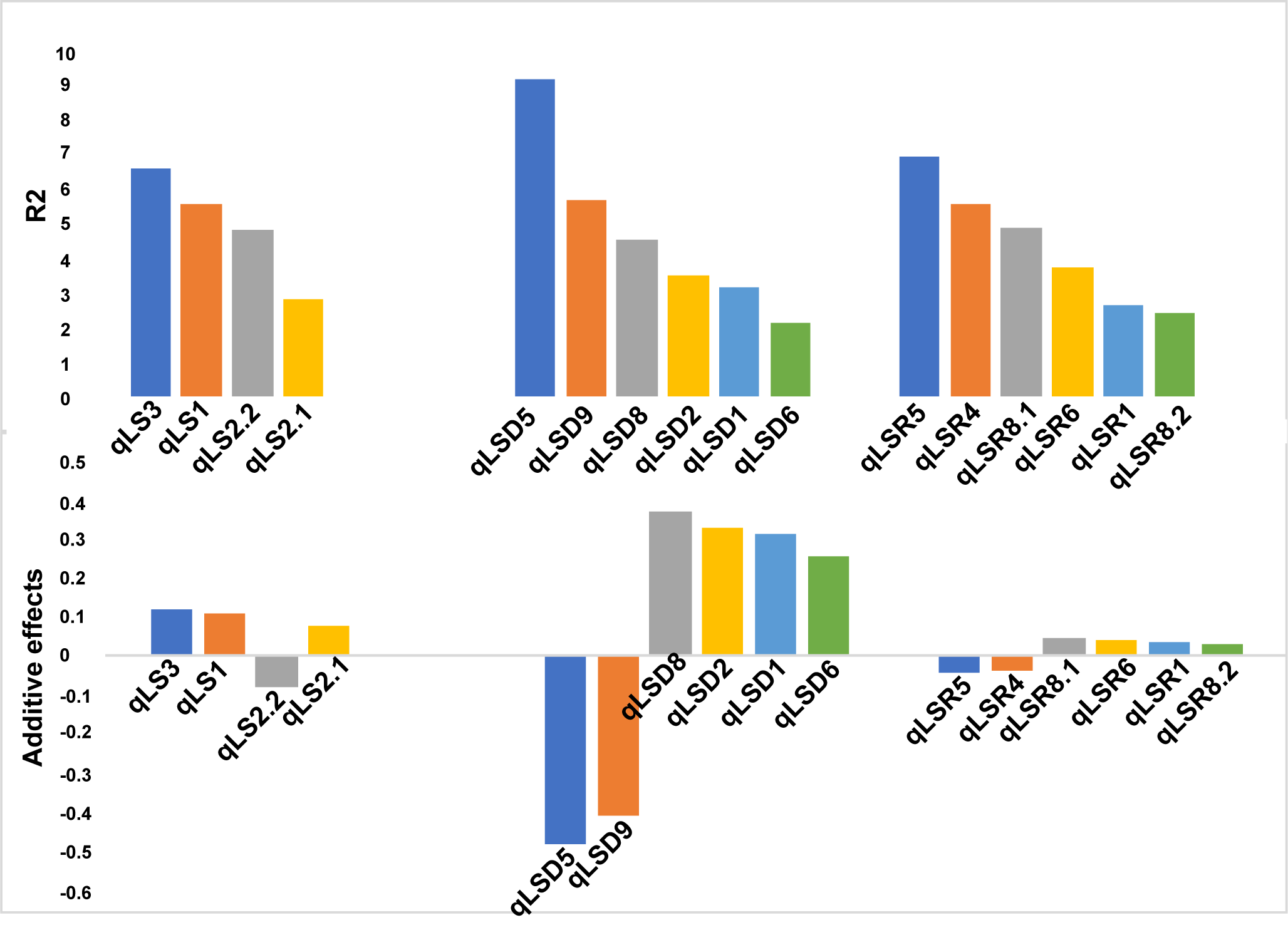

Supplement: Supplementary Figure 7 — The R2 and additive effect of quantitative trait loci (QTLs) for thermotolerance traits in the RIL-F2:8 population. The x-axis indicates each identified QTL, and the y-axis indicates the percentage of phenotypic variance explained by a QTL (up) and additive effects (down) by each QTL. [file Image_7.tif]

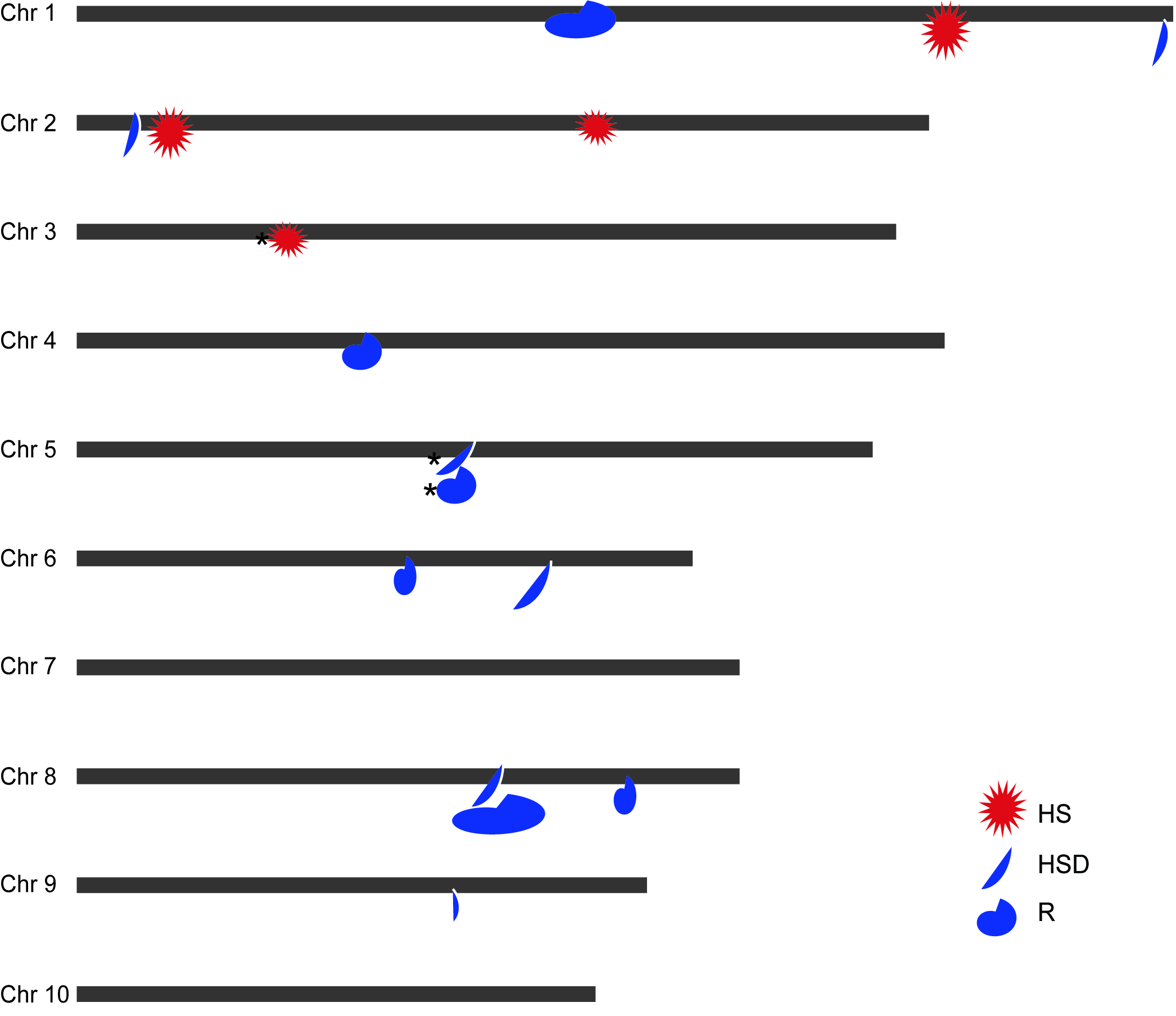

Supplement: Supplementary Figure 8 — Unveiling the chromosomal distribution of QTLs in Maize under high temperature stress during the flowering stage. The larger the graphic of each QTL locus, the larger the genomic region; the narrower the graphic, the smaller the genomic region. [file Image_8.tif]
