# Supplementary material for: A SLAF-based high-density genetic map construction and genetic architecture of thermotolerant traits in maize (Zea mays L.)
Source: Front Plant Sci. 2024 Feb 7;15:1338086. doi: 10.3389/fpls.2024.1338086 (PMC10880447; doi:10.3389/fpls.2024.1338086)
Supplement: Supplementary Table 8 — The thermosensitive phenotypes from RIL-F2:8 population under high temperature stress at flowering in maize. [file DataSheet_1.zip › Data Sheet 1 (20)/Supplemental Table 1 qPCR primer design for candidate genes.docx]

**Supplementary Table S1.** qPCR primer design for candidate genes.

| Gene1_qPCR_F | GAGCTGGTGTTCGTGAACAAGT |
| --- | --- |
| Gene1_qPCR_R | AGCAGCTTTAGGGCCAACTC |
| Gene2_qPCR_F | TGTTGATCTCTCAGCGGACTTC |
| Gene2_qPCR_R | GTTCGGTCAAACCATACACAGC |
| Gene3_qPCR_F | AGTGCAATATGAGCAGCCTCAC |
| Gene3_qPCR_R | AGCTCTCGCTCAGCATCGT |
| Gene4_qPCR_F | TTGATAGACAGGCTCCTCATGG |
| Gene4_qPCR_R | GCAGGGACAATTTCCCAGTATT |
| Gene5_qPCR_F | TACGTGTCGCTGTGCTTGTATC |
| Gene5_qPCR_R | AGCAGCAGCTTTGACACCTT |
| Gene6_qPCR_F | AACGTCTCGAAGCTCCTCAA |
| Gene6_qPCR_R | TGCAGCACGGAGATGTAGG |
| ZmTubulinF | GCGCCTGTCTGTTGACTATGG |
| ZmTubulinR | GGGATGGGTACACGGTGAAA |
