# Supplementary material for: A SLAF-based high-density genetic map construction and genetic architecture of thermotolerant traits in maize (Zea mays L.)
Source: Front Plant Sci. 2024 Feb 7;15:1338086. doi: 10.3389/fpls.2024.1338086 (PMC10880447; doi:10.3389/fpls.2024.1338086)
Supplement: Supplementary Table 8 — The thermosensitive phenotypes from RIL-F2:8 population under high temperature stress at flowering in maize. [file DataSheet_1.zip › Data Sheet 1 (20)/Supplemental Table 10 Phenotypic variation of three thermotolerance traits.docx]

**Supplementary Table 10.** Phenotypic variation of three thermotolerance traits in the F_2:8_ RIL population.

| Trait | Minimum | Maximum | Mean | SD | Variance | Skewness | Kurtosis | H2 |
| --- | --- | --- | --- | --- | --- | --- | --- | --- |
| LS | 1.00 | 3.00 | 2.27 | 0.83 | 0.70 | -0.55 | -1.34 | 0.73 |
| LSD | 1.00 | 9.00 | 3.36 | 1.71 | 2.91 | 0.89 | 0.53 | 0.38 |
| LSR | 0.00 | 0.80 | 0.29 | 0.18 | 0.03 | 0.63 | 0.00 | 0.60 |
