# Supplementary material for: A SLAF-based high-density genetic map construction and genetic architecture of thermotolerant traits in maize (Zea mays L.)
Source: Front Plant Sci. 2024 Feb 7;15:1338086. doi: 10.3389/fpls.2024.1338086 (PMC10880447; doi:10.3389/fpls.2024.1338086)
Supplement: Supplementary Table 8 — The thermosensitive phenotypes from RIL-F2:8 population under high temperature stress at flowering in maize. [file DataSheet_1.zip › Data Sheet 1 (20)/Supplemental Table 2 SLAF marker development based on high-throughput SLAF sequencing data.docx]

**Supplementary Table S2.** SLAF marker development based on high-throughput SLAF sequencing data.

| Sample | SLAF Number | Total Depth (X) | Average Depth(X) |
| --- | --- | --- | --- |
| Abe2 (P) | 684,620 | 12,654,046 | 18.48 |
| B73 (M) | 955,168 | 22,878,332 | 23.95 |
| Offspring | 420,299 | 3,262,227 | 8.05 |

P: paternal inbred line;

M: maternal inbred line.
