# Supplementary material for: A SLAF-based high-density genetic map construction and genetic architecture of thermotolerant traits in maize (Zea mays L.)
Source: Front Plant Sci. 2024 Feb 7;15:1338086. doi: 10.3389/fpls.2024.1338086 (PMC10880447; doi:10.3389/fpls.2024.1338086)
Supplement: Supplementary Table 8 — The thermosensitive phenotypes from RIL-F2:8 population under high temperature stress at flowering in maize. [file DataSheet_1.zip › Data Sheet 1 (20)/Supplemental Table 3 Number of 3 types of SLAF markers.docx]

**Supplementary Table S3.** Number of 3 types of SLAF markers.

| Type | Polymorphic SLAF | Non-Polymorphic SLAF | Repetitive SLAF | Valid marker | Total SLAF |
| --- | --- | --- | --- | --- | --- |
| Number | 108,709 | 480,825 | 236 | 48,397 | 589,770 |
| Percentage | 18.43% | 81.53% | 0.04% | 8.21% | 100% |
