# Supplementary material for: A SLAF-based high-density genetic map construction and genetic architecture of thermotolerant traits in maize (Zea mays L.)
Source: Front Plant Sci. 2024 Feb 7;15:1338086. doi: 10.3389/fpls.2024.1338086 (PMC10880447; doi:10.3389/fpls.2024.1338086)
Supplement: Supplementary Table 8 — The thermosensitive phenotypes from RIL-F2:8 population under high temperature stress at flowering in maize. [file DataSheet_1.zip › Data Sheet 1 (20)/Supplemental Table 4 Distribution of SLAF markers on each linkage group.docx]

**Supplementary Table S4.** Distribution of SLAF markers on each linkage group.

| ChrID | SLAF Number | Polymorphic SLAF |
| --- | --- | --- |
| 1 | 85,192 | 15,154 |
| 2 | 67,059 | 12,513 |
| 3 | 66,886 | 12,730 |
| 4 | 67,713 | 13,381 |
| 5 | 59,588 | 11,345 |
| 6 | 46,174 | 8,284 |
| 7 | 50,638 | 9,958 |
| 8 | 49,626 | 8,820 |
| 9 | 45,649 | 7,321 |
| 10 | 42,872 | 7,825 |
| other | 8,373 | 1,378 |
| Total | 589,770 | 108,709 |
