# Supplementary material for: A SLAF-based high-density genetic map construction and genetic architecture of thermotolerant traits in maize (Zea mays L.)
Source: Front Plant Sci. 2024 Feb 7;15:1338086. doi: 10.3389/fpls.2024.1338086 (PMC10880447; doi:10.3389/fpls.2024.1338086)
Supplement: Supplementary Table 8 — The thermosensitive phenotypes from RIL-F2:8 population under high temperature stress at flowering in maize. [file DataSheet_1.zip › Data Sheet 1 (20)/Supplemental Table 5 The 2-allelic coding rules of genetics.docx]

**Supplementary Table S5.** The 2-allelic coding rules of genetics.

| Type | Paternal genotype | Maternal genotype | Offspring genotype |
| --- | --- | --- | --- |
| abxcd | ab | cd | ac,ad,bc,bd |
| efxeg | ef | eg | ee,ef,eg,fg |
| abxcc | ab | cc | ac,bc |
| ccxab | cc | ab | ac,bc |
| hkxhk | hk | hk | hh,hk,kk |
| lmxll | lm | ll | lm,ll |
| nnxnp | nn | np | nn,np |
| aaxbb | aa | bb | F2(aa,ab,bb), RIL/DH(aa,bb) |
