# Supplementary material for: A SLAF-based high-density genetic map construction and genetic architecture of thermotolerant traits in maize (Zea mays L.)
Source: Front Plant Sci. 2024 Feb 7;15:1338086. doi: 10.3389/fpls.2024.1338086 (PMC10880447; doi:10.3389/fpls.2024.1338086)
Supplement: Supplementary Table 8 — The thermosensitive phenotypes from RIL-F2:8 population under high temperature stress at flowering in maize. [file DataSheet_1.zip › Data Sheet 1 (20)/Supplemental Table 6 Spearman correlation coefficient.docx]

**Supplementary Table S6.** Spearman correlation coefficient between map position of each linkage group and physical location on the genome.

| LG | Spearman |
| --- | --- |
| 1 | 0.999 |
| 2 | 0.999 |
| 3 | 0.999 |
| 5 | 0.997 |
| 6 | 0.999 |
| 7 | 0.993 |
| 8 | 0.994 |
| 4 | 0.989 |
| 9 | 0.997 |
| 10 | 0.993 |
