# Supplementary material for: A SLAF-based high-density genetic map construction and genetic architecture of thermotolerant traits in maize (Zea mays L.)
Source: Front Plant Sci. 2024 Feb 7;15:1338086. doi: 10.3389/fpls.2024.1338086 (PMC10880447; doi:10.3389/fpls.2024.1338086)
Supplement: Supplementary Table 8 — The thermosensitive phenotypes from RIL-F2:8 population under high temperature stress at flowering in maize. [file DataSheet_1.zip › Data Sheet 1 (20)/Supplemental Table 7 Average sequencing depth of the map markers.docx]

**Supplementary Table S7.** Average sequencing depth of the map markers for each linkage group.

| Sample | Marker Number | Total Depth | Average Depth(X) |
| --- | --- | --- | --- |
| L403 | 207,054 | 10,765,191 | 51.99 |
| B73 | 350,175 | 20,474,288 | 58.47 |
| Offspring | 228,146 | 2,819,545 | 12.53 |
