# Supplementary material for: A SLAF-based high-density genetic map construction and genetic architecture of thermotolerant traits in maize (Zea mays L.)
Source: Front Plant Sci. 2024 Feb 7;15:1338086. doi: 10.3389/fpls.2024.1338086 (PMC10880447; doi:10.3389/fpls.2024.1338086)
Supplement: Supplementary Table 8 — The thermosensitive phenotypes from RIL-F2:8 population under high temperature stress at flowering in maize. [file DataSheet_1.zip › Data Sheet 1 (20)/Supplemental Table 9 Partial separation marker of each linkage group map.docx]

**Supplementary Table 9.** Partial separation marker of each linkage group map.

| LG | Number | paternal | maternal |
| --- | --- | --- | --- |
| 1 | 255 | 0 | 255 |
| 2 | 41 | 0 | 41 |
| 3 | 78 | 0 | 78 |
| 4 | 251 | 251 | 0 |
| 6 | 6 | 0 | 6 |
| 7 | 9 | 0 | 9 |
| 8 | 31 | 0 | 31 |
| 9 | 15 | 0 | 15 |
| 10 | 56 | 8 | 48 |
| Total | 742 | 259 | 483 |

LG: linkage group;

Number: the number of partial separation markers.
